# West Nile Virus Infection, Assam, India

**DOI:** 10.3201/eid1705.100479

**Published:** 2011-05

**Authors:** Siraj A. Khan, Prafulla Dutta, Abdul M. Khan, Pritom Chowdhury, Jani Borah, Pabitra Doloi, Jagadish Mahanta

**Affiliations:** Author affiliation: Regional Medical Research Centre, Assam, India

**Keywords:** West Nile virus, Japanese encephalitis virus, India, viruses, neutralizing antibody, clinical symptoms, neurologic sequelae, letter

**To the Editor:** West Nile virus (WNV) is a mosquito-borne flavivirus. Sporadic infections with this virus have been found in Africa, Europe, Asia, and the United States. In humans, most infections with WNV cause subclinical or a mild influenza-like illness; encephalitis occurs in some (*1*). In India, antibodies against WNV were first detected in humans in Bombay in 1952 (*2*). Virus activity has been reported in southern, central, and western India. WNV has been isolated in India from *Culex vishnui* mosquitoes in Andhra Pradesh and Tamil Nadu, from *Cx*. *quinquefasciatus* mosquitoes in Maharashtra, and from humans in Karnataka State (*3*).

Assam (26°–27°30′N, 89°58′–95°41′E) is the most populated state in northeastern India; it contains ≈50% of the 38.8 million inhabitants of northeastern India. Japanese encephalitis virus (JEV) has caused sporadic epidemics in Assam since 1976. Studies conducted during 2000–2002 in Assam showed that 187 (53.7%) of 348 persons with acute encephalitis syndrome were infected with JEV (*4*). JEV-negative persons also showed symptoms of neurotropic viral infection.

Suspecting the presence of some other closely related flavivirus in this region, we screened samples from persons with acute encephalitis syndrome for WNV in 2006. To our knowledge, no study has been conducted on the prevalence of WNV in this region. We report WNV activity in the state of Assam in northeastern India. Ethical approval for this study was obtained from the institutional ethical committee, Regional Medical Research Center, Dibrugarh, India.

A JEV vaccination campaign (SA14-14-2 vaccine) was started in Assam during May 2006. During its first phase, children 1–15 years of age in Dibrugarh and Sivasagar Districts were vaccinated. Mosquito surveillance in the study area and in an earlier study (*5*) identified *Cx*. *vishnui* mosquitoes.

During the study period, 103 serum samples and 88 cerebrospinal fluid samples were obtained from 167 patients with acute encephalitis syndrome admitted to the Assam Medical College and Hospital in Dibrugarh, which administers to the health needs of >7 districts of Upper Assam and neighboring states of Arunachal Pradesh and Nagaland. Among the 167 patients, 124 (74.2%) were children <15 years of age.

Among the 103 serum samples, 80 were positive for immunoglobulin (Ig) M against JEV (IgM monoclonal antibody–capture ELISA; National Institute of Virology, Pune, India) and 12 (11.6%) were positive for IgM against WNV (IgM antigen-capture ELISA; Panbio, Sinnamon Park, Queensland, Australia). These samples were from persons in 4 districts in Assam (Dibrugarh, Golaghat, Sivasagar, and Tinsukia) and negative for IgM against JEV (Table). Follow-up was conducted for 9 patients; 3 died, and 1 was lost to follow-up.

**Table:** Incidence of JEV and WNV infections among patients with acute encephalitis syndrome, Assam, India*

District	No. with acute encephalitis syndrome	No. positive/no. tested	
		JEV	WNV
Dhemaji	1	0/1	0/0
Dibrugarh	29	9/29	6/29
Golaghat	81	47/81	2/18
Jorhat	15	8/15	0/15
Lakhimpur	6	5/6	0/6
Sivasagar	30	9/30	2/30
Tinsukia	5	2/5	2/5
Total	167	80/167	12/103†

*JEV, Japanese encephalitis virus; WNV, West Nile virus. †One person was not included because the address could not be verified.

Virus-neutralizing antibody titers against JEV and WNV were estimated in pig kidney epithelial cells by using JEV (isolate 733913) and WNV (isolate 68856) and a cytopathic-effect assay in 96-well tissue culture plates (*6*). Mouse polyclonal antibodies against JEV and WNV and nonimmune serum samples were included in the assay. Of 9 paired serum samples, 6 showed neutralizing antibody for WNV, of which 4 showed a 4-fold increase in antibody titer. The remaining 3 paired samples showed cross-reactivity with WNV (titer <80) and JEV (titer <40).

All 12 WNV-infected patients had high fever and headache. Convulsions (6 patients), altered sensorium (7 patients), vomiting (5 patients), and neck rigidity (2 patients) were also observed. Signs and symptoms at the time of hospitalization and at follow-up for 6 months (at 3-month intervals) were similar for persons infected with JEV and those infected with WNV. Neurologic sequelae observed at <6 months follow-up were impaired memory (6 patients), irritable behavior (5 patients), impaired hearing (3 patients), incoherent speech and disorientation (1 patient), breathing difficulty (1 patient), impaired speech (1 patient), and quadriparesis (1 patient).

We identified WNV in regions of Assam to which JEV is endemic. The finding indicates that WNV might be the cause of a substantial number of acute encephalitis syndrome cases in this region. Fever and headache were the most common signs and symptoms, as reported (*7*). There were 3 deaths (all children) in 13 patients. Our results corroborate a similar observation in the Kolar District of Karnataka (*8*). In contrast, in western countries, the attack rate and case-fatality rate for WNV infection are higher among immunocompromised elderly patients (*9*). Our findings may be caused by strain variations and host susceptibility to the virus. Identification of circulating genotypes of WNV and its vectors and epidemiologic studies are needed to obtain additional information on WNV infection in this region and identify WNV as a cause of acute encephalitis syndrome.
